# Antioxidant and Antitumor Potential of Micropropagated Balkan Endemic *Sideritis scardica* Griseb

**DOI:** 10.3390/plants12233924

**Published:** 2023-11-21

**Authors:** Krasimira Tasheva, Ani Georgieva, Petko Denev, Lyudmila Dimitrova, Margarita Dimitrova, Svetlana Misheva, Polina Petkova-Kirova, Maria Lazarova, Maria Petrova

**Affiliations:** 1 Department of Plant Ecophysiology, Institute of Plant Physiology and Genetics, Bulgarian Academy of Sciences, Acad. G. Bonchev Str. 21, 1113 Sofia, Bulgaria; dim.lyudmila@gmail.com (L.D.); mstoyadinova@abv.bg (M.D.); s_landjeva@mail.bg (S.M.); 2 Department of Pathology, Institute of Experimental Morphology, Pathology and Anthropology with Museum, Bulgarian Academy of Sciences, Acad. G. Bonchev Str. 21, 1113 Sofia, Bulgaria; georgieva_any@abv.bg; 3 Laboratory of Biologically Active Substances, Institute of Organic Chemistry with Centre of Phytochemistry, Laboratory of Bioactive Substances, Bulgarian Academy of Sciences, 4000 Plovdiv, Bulgaria; petko.denev@orgchm.bas.bg; 4 Department of Synaptic Signaling and Communication, Institute of Neurobiology, Bulgarian Academy of Sciences, 1113 Sofia, Bulgaria; kirovaps@yahoo.com (P.P.-K.); m.lazarova@gmail.com (M.L.)

**Keywords:** Mursala tea (*Sideritis scardica*), multiplication, antioxidant activity, tumor cell line

## Abstract

*Sideritis scardica* Griseb. is a critically endangered Balkan endemic species, known for its antioxidant, neuroprotective and anti-inflammatory properties. The aim of the present study was to detail an efficient protocol for the micropropagation of *S. scardica*. In vitro cultures were initiated from the shoot tips of 40 days-old in vivo seedlings and the effects of different plant growth regulator treatments were examined. A Murashige and Skoog nutrient medium (MS) containing 1 mg/L zeatin and 0.1 mg/L indole-3-acetic acid (IAA) proved to be the most efficient for shoot multiplication as it produced quality, vigorous shoots with a mean number of six shoots per explant. For the first time, the antioxidant and antitumor activities of extracts from in vitro-obtained plants were evaluated. In vitro cultivated plants grown in the field revealed a higher total polyphenol content (3929.1 ± 112.2 mg GAE/100 g vs. 3563.5 ± 52.8 mg GAE/100 g) and higher ORAC antioxidant activity (1211.6 ± 27.3 µmol TE/g vs. 939.9 ± 52.4 µmol TE/g) than in situ cultivated plants. A comparison of the antitumor activities of extracts from in vitro propagated shoots, field-grown in vitro-obtained plants and in situ plants on HeLa (cervical adenocarcinoma), HT-29 (colorectal adenocarcinoma) and MCF-7 (breast cancer) human cancer cell lines showed that in vitro propagated shoots had a significant concentration-dependent cytotoxic effect on the cervical adenocarcinoma cell line HeLa, while the field-grown in vitro-obtained and in situ-collected samples induced the highest reduction in the viability of the mammary carcinoma cell line MCF-7. In both cases, the cells of the control non-tumor cell line, BALB/3T3, were significantly less affected. The results showed that the in vitro multiplication protocol ensured the obtainment of numerous plants with antioxidant and antitumor potential.

## 1. Introduction

In recent years, attention has been largely focused on the problem of preserving plant biodiversity endangered by habitat destruction and ecological fragmentation, the uncontrolled collection of wild plants, inappropriate agricultural and forestry practices, urbanization and pollution [1]. Special consideration is given to the conservation of the gene pool of plant species with a high rate of extinction, not only to maintain botanical heritage, but also to preserve rare plants with valuable, and sometimes unique, medicinal properties [2,3]. Biotechnology is especially useful for plant genetic preservation, as well as for obtaining biomass and further plant secondary metabolites under controlled conditions, particularly from vulnerable, imperiled species. Using effective in vitro propagation techniques, it is possible to obtain both an unlimited number of plants and constant metabolite content in the culture [4].

*Sideritis scardica* Griseb (commonly known as Mursala tea, Pirin tea or Alibotush tea) is a Balkan endemic species listed as critically endangered in the Red List of Bulgaria, with a complete ban on its collection from its natural habitats, and assessed as Near Threatened by the IUCN Global Red List [5]. It belongs to the Lamiaceae family and is one of the three largest genera of the family, which include more than 150 species, found mostly in the Mediterranean region [6]. In Bulgaria, part of the *S. scardica* populations are within the Alibotush Strict Nature Reserve (Slavyanka Mt.), Pirin National Park and Trigradsko Zdrelo Protected Site (Rhodope Mts.) [7]. The cultivation of the species is very important to preserve its limited distribution. The species is grown as a cultivated plant (in situ) in its natural habitat, Trigrad, Rhodope Mountains. The aerial parts of the species have been used in traditional medicine for many years to treat common colds and gastrointestinal disorders [8,9]. *Sideritis* spp. are rich in unique bioactive secondary metabolites, including terpenes, sterols, coumarins, flavonoids, phenols, iridoids and lignins, and over 160 diterpenes [10]. Sideritis metabolites reveal antioxidant [11,12,13,14,15], antimicrobial [16,17,18], anti-inflammatory [19], neuroprotective [20,21,22] and other properties. In recent years, the antitumor activity of the species has also been investigated in relation to various types of cancer, such as liver and colon cancer [19,23,24,25,26,27]. The high concentration of phenolic compounds, and especially of some flavonoids, is one reason for its cytotoxic effect against cancer cells [19,27,28]. An antitumor effect of siderol (diterpenoid) has been demonstrated in glioblastoma GBM T98 and U87 cell lines [29] and in C6 rat glioma cells [23].

Despite the great interest in *S. scardica* and the extensive research on its phytochemical composition, elaborate in vitro methods for its cultivation are very limited. In the literature, there are only a few reports on the in vitro propagation of *Sideritis scardica* [30,31,32] compared to the numerous reports on other *Sideritis* species [33,34,35,36,37,38]. The in vitro cultivation of *S. scardica* has been shown to be especially challenging, being dependent on a number of different factors and conditions [31].

Thus, the aim of the present study was to develop and detail an efficient protocol for the in vitro propagation of the endangered *Sideritis scardica* Griseb. and to evaluate and compare the antioxidant and antitumor activity of the in vitro-obtained and in situ-cultivated plants.

## 2. Results

### 2.1. In Vitro Culture

#### 2.1.1. Shoot Induction and Multiplication

**Figure 1:**
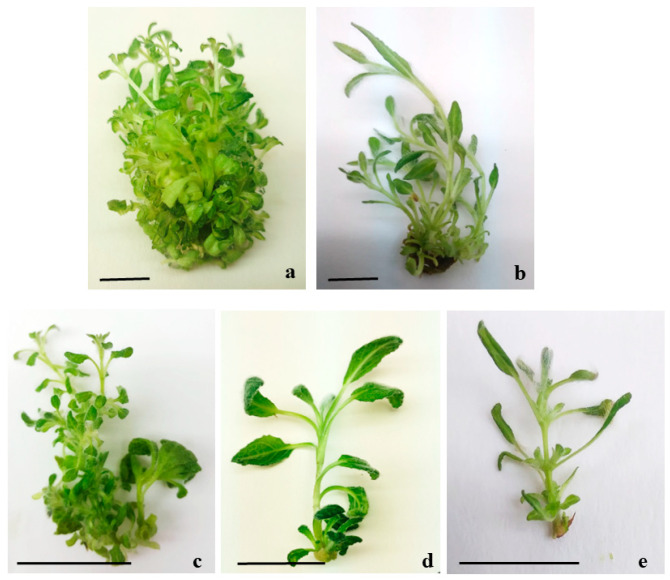
Shoot multiplication of *S. scardica* on MS medium with different composition (**a**) Z_2_I_0.1_ (**b**) Z_1_I_0.1_ (**c**) B_1_I_0.1_ (**d**) K_1_N_0.1_ (**e**) T_1_N_0.1_. Scale bar represents: (**a**,**b**,**d**) = 0.5 cm; (**c**,**e**) = 0.25 cm.

The most effective seed disinfection method was found to be soaking the seeds in ethanol for 2 min followed by mercuric chloride solution for 15 min. The other examined disinfection treatments were not effective, and bacterial contamination was noticed. Despite the successful disinfection, no seed germination was observed in the tested culture period. The in vitro culture initiation was achieved using stem tips excised from seedlings and raised in in vivo conditions. The stem explants grown on basic Murashige and Skoog (MS) medium without plant growth regulators (PGRs) did not produce new shoots within four weeks of culture. At the end of the cultivation period, necrosis of the tips of some shoots grown on the control medium was observed. All of the PGRs treatments increased the multiplication potential significantly. The morphogenetic response of explants was influenced by the type and concentration of cytokinins and auxins used (Table 1; Figure 1). The frequency of shoot multiplication varied between 60 and 100%. Among the examined nutrient media, the best results regarding the number of shoots per explant and the quality of shoots were achieved on zeatin-containing media. Most of the shoots (mean number of 11 per explant) were produced on MS medium enriched with 2 mg/L zeatin and 0.1 mg/L indole-3-acetic acid (IAA), although 10% of them were hyperhydrated (Figure 1a). The nutrient medium containing 1 mg/L zeatin and 0.1 mg/L IAA was chosen as the best one for shoot multiplication as it produced quality, vigorous shoots, with a mean number of 6 shoots per explant (Figure 1b). In addition, the shoot elongation was high (2.4 cm). The nutrient medium supplemented with 1 mg/L 6-benzylaminopurine (BAP) and IAA also formed 6.0 shoots per explant; however, 15% of them were hyperhydrated (Figure 1c). The medium augmented with 1 mg/L BAP and 0.1 mg/L IAA was more efficient for shoot proliferation compared to the combination of 1 mg/L BAP and 0.1 mg/L α-naphthalene acetic acid (NAA). Most of the tested PGRs induced new shoots at the base of the explant. The media supplemented with kinetin (K_1_I_0.1_ and K_1_N_0.1_) formed an average of 4–5 shoots per explant, but the height of the shoots was low (Figure 1d). Only in the thidiazuron-containing medium (1 mg/L thidiazuron and 0.1 mg/L NAA), shoots grew from the node area of the stem explants (Figure 1e). In the subsequent subcultures, the new shoots, when cut into segments containing one or two nodes and cultured in Z_1_I_0.1_ medium, gave rise to multiple new shoots. The number of shoots increased for the subsequent 3–4 subcultures, and decreased thereafter. 

#### 2.1.2. In Vitro Rooting and Acclimatization 

**Figure 2:**
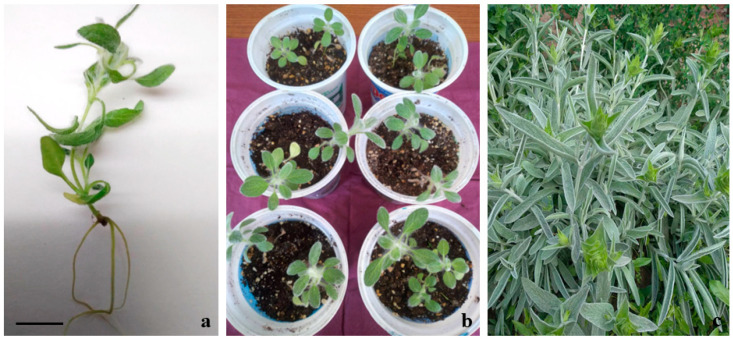
In vitro rooting and acclimation of *S. scardica* (**a**) in vitro rooted plant on half strength MS medium supplemented with 0.6 mg/L IAA. Scale bar represents: 1.0 cm (**b**) ex vitro adapted plants (**c**) plants during flowering stage.

The rooting stage was an important step in the micropropagation protocol. The well-developed and healthy root system ensured a high survival rate during acclimatization. Shoots cultured on the control half strength MS medium without PGRs treatment failed to induce roots. The rhizogenesis of the in vitro-obtained micropropagated shoots required the addition of auxin to the culture medium. The shoots formed roots on all of the examined nutrient media containing different IAA concentrations; however, the frequency of root development was low, at about 15–60% (Table 2). Root primordia from the basal end of the shoots were noticed 10 days after transfer to the rooting media. The best nutrient medium for in vitro rooting was the half-strength MS medium supplemented with 0.6 mg/L IAA, which resulted in the highest number of roots per shoot with the largest root elongation (Figure 2a). The gradual exposure of the in vitro-rooted plants to ex vitro conditions ensured their adaptation. The substrate composition on which the in vitro plantlets were adapted contained peat, perlite, sand and soil in a ratio of (2:1:1:1) and ensured an up to 70% survival rate. The plants were hardened within a period of 1 month in greenhouse. No morphological alterations were observed among the ex vitro-adapted plants (Figure 2b,c). The plants were successfully adapted in “Elin Pelin” fields. Most of the plants (80%) bloomed in the second year.

### 2.2. Content of Total Polyphenols, Total Flavonoids and Antioxidant Activity

The total polyphenol and flavonoid contents, as well as the antioxidant activity, of in vitro propagated shoots, field-grown in vitro-obtained plants and in situ plants are shown in Table 3. From the results, it is evident that the field-grown in vitro-obtained plants accumulated the highest amounts of total polyphenols and flavonoids. The amount of polyphenols varied between 2404.6 ± 30.7 mg gallic acid equivalents (GAE)/100 g dry weight (DW) for the in vitro shoots and 3929.1 ± 112.2 mg GAE/100 g DW for the in vitro-obtained field grown plants. The content of flavonoids in the in situ-collected samples and field-grown plants was similar, although it was slightly higher in the latter, at 1024.3 ± 31.1 mg rutin equivalents (RE)/100 g DW. The higher content of polyphenols and flavonoids in the field-grown plants renders them as possessing higher antioxidant activity, assessed using both the hydroxyl radical averting capacity (HORAC) (307.7 µmol GAE/g) and oxygen radical absorbance capacity (ORAC) (1211.6 ± 27.3 TE/g) methods. The in vitro-propagated shoots had the lowest content of total polyphenols and flavonoids and revealed the lowest antioxidant activity.

### 2.3. Antitumor Activity

#### 2.3.1. Cell Viability Assay

**Figure 3:**
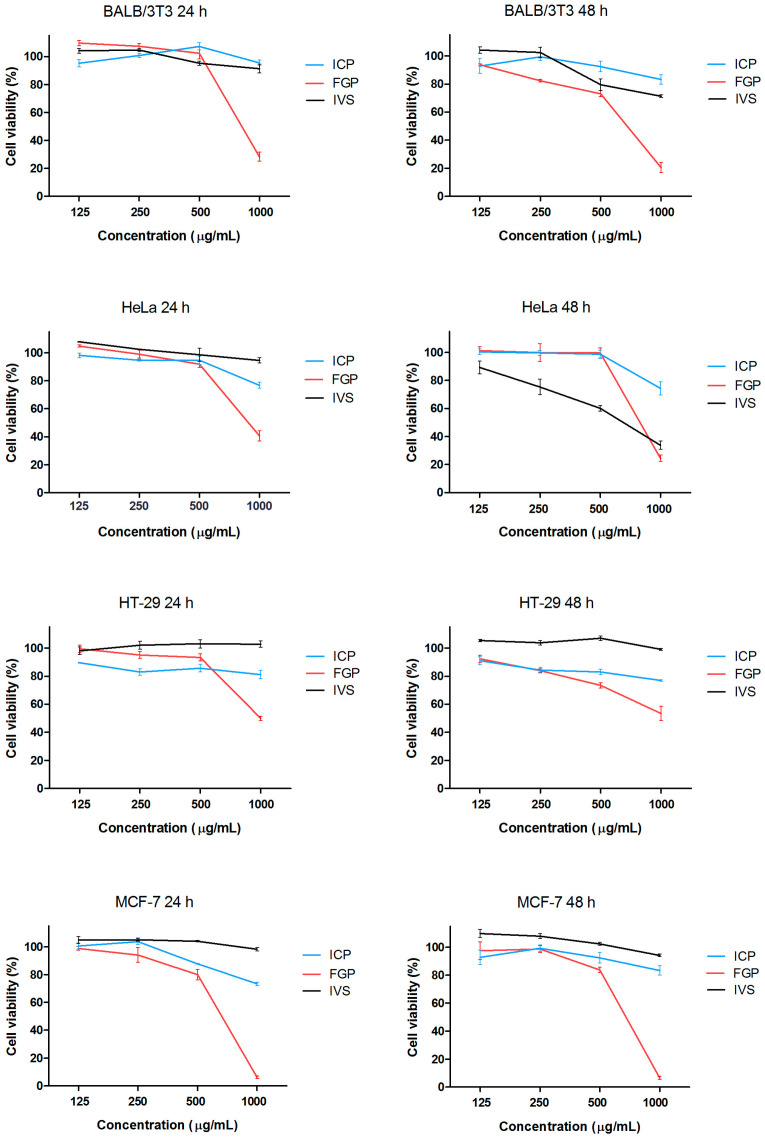
Effects of *Sideritis scardica* plant extracts on the viability and proliferation activity of tumor and non-tumor cell lines assessed through the MTT test after 24 and 48 h of exposure. ICP—in situ-cultivated plants; FGP—in vitro-obtained, field-grown plants; IVS—in vitro-propagated shoots.

The effects of the studied *Sideritis scardica* plant extracts on the viability and proliferation of the BALB/3T3, HeLa, HT-29 and MCF-7 cell lines were examined using the MTT assay after 24 and 48 h of treatment. The concentration–response curves of the extracts tested on tumor and non-tumor cells are presented in Figure 3.

As is evident from the presented data, the FGP extract showed the highest cytotoxic activity among all of the tested cell lines. The viability of the non-tumor BALB/3T3 cells treated with this extract for 24 h was only significantly reduced at the highest concentration, while at 48 h, significant cytotoxic effects were established at all concentrations higher than 125 µg/mL. In the HeLa cell line, a statistically significant reduction in the cell viability was found at concentrations of 500 and 1000 µg/mL, while in the HT-29 cells, all of the tested concentrations induced a low but statistically significant decrease in the cell viability. The highest cytotoxic activity of the FGP and ICP extracts was established for the MCF-7 cell line and the viability of the cell cultures treated with 1000 µg/mL for 48 h was 6.5 ± 0.6% and 9.8 ± 1.1%, respectively. The viability values of the non-tumor cell exposed to FGP and ICP at the same concentration and time period were 20.6 ± 3.6% and 74.3 ± 3.1%. The effect of the IVS extract on the viability of the non-tumorigenic BALB/3T3 cells was comparable to that of the ICP extract. Regarding the tumor cells, IVS demonstrated significantly lower cytotoxicity compared to FGP and ICP in all of the cell lines tested at 24 h. However, after 48 h of treatment, its effect on the HeLa cells’ viability was markedly increased and even exceeded those of the other two extracts. Based on the presented dose–response curves, the 50% inhibitory concentrations (IC_50_) of the tested extracts were calculated for all of the cell lines used (Table 4).

The cervical carcinoma cells, HeLa, showed the highest sensitivity towards the IVS extract, with an IC_50_ value of 629.2 µg/mL determined after 48 h of treatment. The lowest IC_50_ values of ICP and FGP were established after the 24 h treatment of the MCF-7 cells, at 709.1 and 633.5 µg/mL, respectively. 

#### 2.3.2. Cytomorphological Analysis

**Figure 4:**
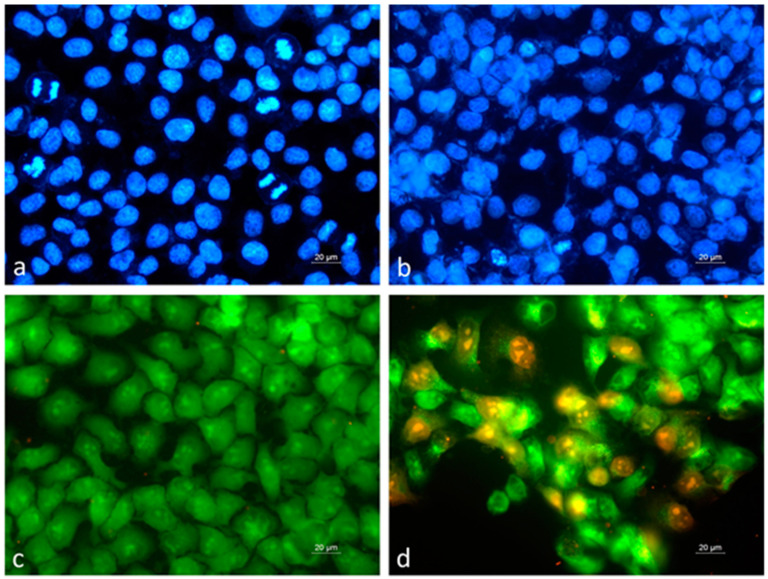
Alterations in the cellular and nuclear morphology of HeLa cervical carcinoma cells induced by extract obtained from in vitro-cultivated *Sideritis scardica* shoots. (**a**,**c**) control untreated HeLa cells; (**b**,**d**) HeLa cells treated with IVS extract; (**a**,**b**) DAPI staining; (**c**,**d**) AO/EB staining.

The alterations in the cellular and nuclear morphology of the HeLa cervical carcinoma cells exposed to the extract obtained from the in vitro-cultivated *S. scardica* shoots for 48 h at a concentration equal to the IC_50_ value determined by the MTT test are shown in Figure 4.

The control untreated cells showed the typical morphological characteristics of this cell line (Figure 4a,c), and numerous dividing cells in different phases of the mitotic cycle were visualized through staining with 4′,6-diamidino-2-phenylindole (DAPI). Acridine orange/ethidium bromide (AO/EB) fluorescent staining of the control HeLa cell cultures revealed a homogenous green fluorescent signal, characteristic of viable cells. The exposure to the IVS extract induced marked alterations in the cellular and nuclear morphology, as presented in (Figure 4b,d). The number of mitotic cells in the IVS-treated cell cultures was significantly reduced, and cells with the morphological features of early and late apoptosis and necrosis were detected.

## 3. Discussion

Due to the protected status and high pharmaceutical value of the Balkan endemic species *S. scardica*, many efforts have been directed to studying the growth biology of the species [7]. In nature, the plant mainly reproduces through seeds, while under cultivation, vegetative propagation through rhizome division is also possible [39].

Biotechnological approaches, including micropropagation methods, are widely used to overcome reproductive difficulties in rare and threatened species, as well as whenever there is a high demand for plant material for the pharmaceutical industry. Multiple shoot inductions through direct organogenesis is the most appropriate method for multiplication, and this carries a lower risk of genetic variability compared to other regeneration methods. It provides repeatedly appearing and abundant saplings in a short time throughout the whole year, irrespective of climate change [40].

In our study, we describe an improved method for the shoot multiplication and ex vitro adaptation of *S. scardica*. For the first time, a protocol for in vitro multiplication has been developed which uses explants from in vivo-grown plants.

Two methods for in vitro culture initiation were used: through seeds and using shoot tips from greenhouse-germinated seedlings. The aseptic seeds failed to germinate in in vitro conditions. The seeds did not achieve germination in our protocols, regardless of whether scarificated or not, and they were not affected by the pre-treatment with gibberellic acid (GA_3_) or the addition of the substance to the nutrient medium. Danova et al. [30] also reported very low germination (1%) under in vitro conditions of *S. scardica* seeds collected from the Slavyanka Mountain (Bulgaria), while a report by Shtereva et al. [31] demonstrated high in vitro seed germination on MS with GA_3_. The germination of seeds of *S. scardica* under natural conditions is also poor [41]. Studies on the seed germination of several *Sideritis* species [42,43] have revealed a strong correlation between germination and environmental conditions.

The successful initiation of in vitro *S. scardica* culture was established using shoot tips from greenhouse-germinated seedlings. The control medium was not suitable for axillary shoot production, and shoot tip necrosis was observed. In previous studies, it was found that PGRs free media require more frequent subculture due to leakage of phenolic substances into the media and browning and necrosis of the plantlets [30]. Thus, the MS medium was altered through the addition of PGRs to achieve multiple and high-quality shoot production. The optimum nutrient medium for micropropagation proved to be MS containing 1 mg/L zeatin and 0.1 mg/L IAA, which yielded high quality shoots and plantlets suitable for subsequent experiments of root induction and ex vitro adaptation. A higher concentration of zeatin (2 mg/L) resulted in multiple shoots, but some of them hyperhydrated. Shtereva et al. [31] likewise used a protocol for the *S. scardica* micropropagation of apical buds and internode stem explants using zeatin-containing nutrient media: MS supplemented with 2 mg/L zeatin and 0.2 mg/L IAA and MS augmented with 0.25 mg/L zeatin and 0.2 mg/L IAA. However, they observed a significantly smaller number of shoots per explant (mean number of three shoots/explant) compared to our protocol, where the average number of newly formed buds on the zeatin-containing media was 6.00–11.00. The data in the literature confirm that the effect of zeatin on plant morphogenesis and shoot formation is highly dependent on its concentration [44]. As the data in the literature show that the combination of BAP (0.2 mg/L and 0.5 mg/L) with NAA (1 mg/L) has a positive effect on the morphological development and polyphenolic production of *S. scardica* in vitro cultures [30], we also studied the effect of the addition of BAP. However, BAP 1 mg/L with IAA 0.1 mg/L resulted only in the formation of buds (height up to 0.5 cm), whereas BAP 1 mg/L with NAA 0.1 mg/L resulted in shoots, albeit lower in number compared to the addition of zeatin in the media. Additionally, the buds and shoots on the media supplemented with BAP in the biggest part were hyperhydrated. Indeed, reports in the literature confirm that even low BAP concentrations lead to hyperhydricity in plant tissue through the subcultivation of *Sideritis athoa* [45]. Moreover, BAP has a lower organogenic potential than zeatin [46]. Because cytokinin-degrading enzymes have a lower affinity for zeatin, zeatin can be present in relatively higher concentrations and has a greater potential for organogenesis in plants than any other cytokinin at the same concentration. This may explain why zeatin has a stimulating effect during shoot regeneration [47]. The positive effect of activated charcoal on axillary shoot development, the compactness of shoots, rooting stimulation and leaf trichome formation was recently reported [32]. 

Regarding rooting, Shtereva et al. [31] achieved a high percentage of rhizogenesis of *S. scardica* on ½ MS supplemented with 2.0 mg/L IBA, 0.2 mg/L IAA and 0.5 mg/L GA_3_. However, Bertsouklis et al. [6] reported that the addition of IBA to the nutrient media reduces the rooting efficiency of *S. raeseri* (14%). Thus, we tested different concentrations of IAA on ½ MS. The best combination was found to be ½ MS supplemented with 0.6 or 1.5 mg/L IAA. 

For an in vitro propagation system to be considered effective, the acclimatization and adaptation of the in vitro-obtained plants in the field should be successful [48]. In our experiments, the acclimatization using a combination of different substrates and under high humidity in the first weeks of adaptation was efficient. The control humidity being crucial for the process is in line with reports by other authors [6,49].

The content of the basal medium and the composition and concentrations of the PGRs used are two parameters that affect the biosynthesis and accumulation of secondary metabolites in in vitro cultures [30]. A number of studies have indicated that shoot culture systems are a source for the production of a range of flavonoids as they have high organogenic potential [50]. *Sideritis* species from the Balkan Peninsula have been studied and were found to be rich in polyphenols and flavonoids that exert antioxidant activity [51]. In order to assess the potential of the in vitro-grown plants to produce secondary metabolites, their polyphenol and flavonoid contents were analyzed and compared with those of plants cultivated in their natural habitat. The field-grown in vitro-obtained plants *S. scardica* were richer in both polyphenols and flavonoids compared to the in situ plants and in vitro-propagated shoots. It is known that the accumulation of phenolic compounds in *S. scardica* could be influenced by different genetic and environmental factors. For example, Evstatieva [52] found the highest content of flavonoids in the budding phase (0.34%), and the lowest content during seed formation (0.02%), with the most flavonoids and tannins found in the leaves and stems, respectively. Later, Evstatieva and Alipieva [53] reported that the content of phenolics and flavonoids was affected by altitude, with a lower altitude leading to a decrease in their content. The differences in the content of phenols and flavonoids between the in vitro-propagated shoots and field-grown in vitro-obtained plants can be attributed to the different growth conditions and stages of plant development [54]. Other factors that could affect the results for the total polyphenol and flavonoid contents are related to the extraction method and the analytical procedure used. For example, quantitative analysis of total phenolics, flavonoids and tannins in different extracts (ethanol, diethyl ether, ethyl acetate, n-butanol) of *S. scardica* from Shara Mountain revealed that ethyl acetate and n-butanolic extracts contain the highest amount of phenolics and flavonoids [19]. In addition, the effect of the extraction method (maceration, ultrasound- and microwave-assisted extraction) from *S. scardica* (cultivar Sofia 2) on the quantitative extraction of secondary metabolites was studied [55]. The extraction effectiveness was evaluated according to the yield of extractives and the yield of total phenolics and total flavonoids. Additionally, extraction through maceration with ethanol and water/ethanol in different proportions and the extraction kinetics were examined [56]. The maximum content of polyphenols and flavonoids was obtained when the H_2_O/EtOH ratio was 20:80. In our study, the amount of polyphenols varied from 2404.6 ± 30.7 mg GAE/100g DW (for in vitro shoots) to 3929.1 ± 112.2 mg GAE/100 g DW for the field-grown in vitro-obtained plants. The content of flavonoids in the in situ plants and field-grown in vitro-obtained plants was similar, although it was slightly higher in the latter, at 1024.3 ± 31.1 mg RE/100g DW. Karapandzova et al. [14] found slightly higher levels of total phenols (47.5 ± 0.6 to 50.8 ± 5.0 mg GA/g) regardless of the habitat and year of collection. The results for the total polyphenols and total flavonoids of Dobrikova et al. [28] for ex situ plants were also higher (59.15 ± 0.92 mg GA/g DW and 21.18 ± 2.12 mg RE/g DW); however, they were still comparable to our results. Yanchev et al. [57] reported that the amount of polyphenols extracted via different extraction procedures (decotion, maceration, ultrasonic and microwave extraction) varied significantly (from 4.0 to 32.2 mg GAE/g dry material). The extraction procedure also affected the amount of flavonoids extracted from the aerial parts of the plant, which were in the range of 1.33 and 5.14 mg RE/g dry material. Regardless of the extraction method, all of the results for the total polyphenol and flavonoid contents in the latter study were lower than the results for the in situ and field-grown in vitro-obtained plants in our study. Danova et al. [32] identified two phenylethanoids and five flavone glycosides in *S. scardica* shoot culture that were not detected in the wild-collected plant material. The authors established that PGRs and activated charcoal selectively affect flavone glycosides and phenylethanoid derivative production. In the literature, there are several reports on the antioxidant activity of *S. scardica* extracts from various locations, obtained under different extraction conditions or analyzed using different antioxidant assays (DPPH, ABTS, ORAC) [58]. Using the ORAC assay, it was revealed that *S. scardica* possesses moderate antioxidant activity when compared to other Bulgarian herbs [59]. Using the FRAP method, it was shown that microwave-assisted extracts obtained using 70% ethanol revealed higher activity [57], whereas another study revealed that butanol, ethyl acetate and the total methanol extracts showed strong radical scavenging activity against DPPH close to that of rosmarinic acid (94.5%) [60]. Dobrikova et al. [28] reported DPPH and FRAP activities of 71.75 ± 1.09 μmol TE/g DW and 77.50 ± 1.10 μmol Fe^2+^/g DW, respectively, for hydroethanolic extract. Karapandzova et al. [14] reported DPPH radical scavenging activity with IC_50_ values between 3.2 and 8.9 mg/mL for ethanol extracts of *S. scardica* from different localities (North Macedonia and Albania). Furthermore, relatively high antioxidant activity measured using the TEAC method was reported by Ivanova et al. [61]. Another study observed significant variation among the measured values in FRAP activity, ranging from 17.04 to 118.75 μM Fe^2+^g^−1^ DW between eight ecotypes of *S. scardica* from different regions of Bulgaria [62]. To the best of our knowledge, the current study is the first report on the evaluation of ORAC and HORAC antioxidant activities of in vitro propagated shoots and field-grown in vitro-obtained plants of *S. scardica*. The differences in total polyphenols rendered different ORAC and HORAC values of the investigated samples, and the field-grown in vitro-obtained plants revealed higher antioxidant activity in both assays. The current study indicates that the elaborated protocol for the in vitro propagation of *S. scardica* produces plants that accumulate comparable amounts of polyphenols, including flavonoids, as those reported for wild and cultivated plants [58]. Moreover, the field-grown in vitro-obtained plants exceeded the polyphenol production of plants that are cultivated in their natural habitat, which is most probably due to environmental and agro technological factors. The higher content of polyphenols and flavonoids rendered the higher antioxidant activity of the field-grown in vitro-obtained plants, suggesting their possible use in oxidative stress-related diseases. 

The use of natural products of plant origin to aid the therapy of certain malignancies has been applied for decades. The trend of using total plant extracts is well grounded, as the pharmacological (protective) effect is most often the result of the synergistic action of the active substances, with their specific mechanisms of action being variable and dependent on their structure and concentration, as well as on various physicochemical environmental factors [63].

Subsequently, the antitumor activities of total aqueous extracts of in vitro propagated shoots, field-grown in vitro-obtained plants and in situ-cultivated plants were compared. The extract from the in situ-cultivated plants showed the strongest cytotoxic effect at 1000 µg/mL, at 24 and 48 h on the MCF-7 cell line, while the viability of the control non-tumorigenic cell line BALB/3T3 was not significantly reduced. Although it also demonstrated effects on the HeLa and HT-29 cell lines, the extract from the in vitro-obtained field-grown plants showed the most significant cytotoxic effects on the MCF-7 line, similar and even slightly higher compared to the effect of that extract from the in situ-cultivated plants. The extract had lower cytotoxic effects on the cells from the non-tumor cell line (which, however, remained significant at 1000 µg/mL). The effect of the extracts from the in vitro-propagated shoots was already evident at a concentration of 250 µg/mL in the HeLa line, with 100% survival of the normal fibroblast cells at this concentration. With an increase in the concentration, the cytotoxic effect increased as well, reaching more than 60% at 1000 µg/mL at 48 h, with no increase in the cytotoxic effect in the non-tumor cell line observed. Moreover, the treatment with IVS extract induced marked changes in the tumor cell morphology that indicated its apoptosis-inducing ability. Thus, extracts from in vitro-propagated plants could prove to be an efficient supporting therapy for cervical cancer (HeLa cell line). Our data for the antitumor activity of extracts of *S. scardica* are in line with the data in the literature, although the literature data are limited to cultured plants, as, to the best of our knowledge, our results are the first to report the antitumor activity of in vitro propagated shoots in comparison with in situ cultured plants. Examining cultured *S. scardica*, Dobrikova et al. [28] found that increasing the concentrations (200, 400 and 600 μg mL^−1^) of the extract, after 24 h of treatment, result in changes in the Colon 26 cell morphology, without affecting the healthy MDCK cells. Concentrations of the extract up to 600 μg mL^−1^ result in a decrease in cell viability in the colon 26 cell line to as low as 40% cell survival. These results suggest a cell-specific antitumor effect of *S. scardica* extracts and are consistent with other studies [19] which report a toxic effect of ethanolic extracts of the plant on melanoma B16 and leukemia HL-60 cells (also very weak) and no cytotoxic effect on human peripheral blood mononuclear cells (PBMC). In contrast to the aqueous extracts used in our study, the diethyl ether and ethyl acetate extracts of *S. scardica* showed significant cytotoxicity against rat C6 glioma cells, but were not cytotoxic on rat astrocytes in primary culture used as healthy control cells [23]. The diethyl ether extracts induced apoptosis and autophagy, whereas the ethyl acetate extracts induced G2/M cell cycle arrest and autophagy.

According to a number of authors, the high concentrations of polyphenols, and especially of some flavonoids, in *S. scardica* are the main reason for its cytotoxic effect, especially on cancer cells [19,23]. The main function of those substances in plants is to counteract stress caused mostly by environmental factors. The protective effect of polyphenols has been observed using many in vitro assays, in vivo models and in humans. In vitro studies strongly suggest the anticancer effects of polyphenols [64]. According to Żyżelewicz et al. [27], the high antioxidant potential of *S. scardica* is attributed to phenolic acids, responsible for protection against oxidative damage, which also underlie carcinogenesis. According to Abotaleb et al. [65], phenolic compounds are robust candidates for the treatment of various cancers. However, even though the anticancer efficacy of individual phytochemicals is supported by multiple lines of evidence, the authors believe that, due to the presence of a mixture of phytochemicals, the whole plant (or total extracts from it) exhibits much more superior anticancer activity than its individual phytocomponents.

## 4. Materials and Methods

### 4.1. In Vitro Culture

#### 4.1.1. Initial Plant Material

The seeds for in vitro experiments were gathered from an ex-situ collection of the Institute of Plant Physiology and Genetics, Bulgarian Academy of Sciences. The plants in the ex-situ collection belong to the Sofia 2 cultivar described by Evstatieva [66].

#### 4.1.2. Sterilization of Plant Material

Seeds and stem tips from 40 days-old in vivo seedlings were used as a starting plant material. Seventy seeds were placed in sterilized soil substrate containing soil, peat, perlite and sand in proportion (1:1:1:1). About 65% of them were germinated and developed into seedlings. For each sterilization treatment, 50 seeds and 46 stem tips were tested.

Seeds were disinfected by applying different procedures: consecutive soaking into 70% (*w*/*v*) ethanol for 2 min, 0.1% (*w*/*v*) mercuric chloride (HgCl_2_) solution for 5, 10 or 15 min. The other procedures included using 70% (*w*/*v*) ethanol for 1 to 3 min and commercial 50% (*w*/*v*) bleach solution for 5, 10 or 15 min. Tween 20 (Sigma-Aldrich, St. Louis, MO, USA), used as an emulsifier, was added in the sterilizing solution. In the subsequent step, the seeds were rinsed three times with autoclaved distilled water to remove traces of the sterilizing agents. Next, the seeds were placed on basic Murashige and Skoog (MS) [67] medium supplemented with 30 g/L sucrose in Petri dishes (6 cm). To stimulate in vitro seeds germination, decontaminated seeds were treated with 10 mg/L GA_3_ for 24 h or were subjected to scarification prior to culturing. Then, the seeds were cultured on MS medium free of PGRs or MS medium containing 10 mg/L GA_3_.

Stem tips (2–3 cm) were excised from 40 days-old seedlings, grown in a greenhouse. They were surface sterilized using HgCl_2_ 0.04% (*w*/*v*) for 20 min, followed by three rinses in sterile distilled water. The bases of the explants were cut off to remove parts of the explants that were damaged in the disinfection procedure. The sterilized explants were inoculated vertically on half strength MS nutrient medium supplemented with 0.5 mg/L IAA and 0.1 mg/L GA_3_.

#### 4.1.3. Media Composition for In Vitro Micropropagation

Stem explants containing nodes were used for the micropropagation experiments. For in vitro multiplication, seven different MS based media, modified for shoot formation and development, were examined. The effect of cytokinins—zeatin (Z) (a mixed isomere) (Sigma-Aldrich Inc., USA), 6-benzylaminopurine (BAP), kinetin (K) or thidiazuron (T)—at concentrations 0.5 or 1.0 mg/L combined with two auxins—α-naphthalene acetic acid (NAA) or indole-3-acetic acid (IAA)—at a concentration 0.1 mg/L (Duchefa Biochemie B.V, Haarlem, The Netherlands) was tested (Table 1). An MS medium without any plant growth regulators was used as a control treatment. All media contained 3% sucrose and were solidified with 0.7% agar (*w*/*v*). The pH of the media was adjusted to 5.8 using NaOH and HCl. All culture media were autoclaved at 1.1 kg cm^−2^, 121 °C, for 20 min.

Shoots were maintained through regular subculture at a four-week culture period on fresh medium with the same composition. Parameters such as the percentage of shoot induction, average number of shoots per explant and length of adventitious shoots were measured.

#### 4.1.4. In Vitro Rooting and Acclimatization of Obtained Plants

For in vitro root induction, multiple shoot clumps were separated and single shoots (1 to 2 cm length) were cultured on half strength nutrient MS medium supplemented with different concentrations of IAA. The nutrient medium supplemented with 0.5 mg/L IAA also contained 0.1 mg/L GA_3_. After four weeks on rooting media, the rooted shoots, after being gently washed with tap water, were transferred to plastic pots (8 cm in diameter) and filled with soil, peat, perlite and sand (2:1:1:1). The pots were ensured to provide high relative humidity for the plants, being enclosed (shielded) for two weeks with clear plastic boxes. Further culturing conditions included 25 °C, 16/8 photoperiod and 50 μM m^−2^ s^−1^ illumination. Five weeks after adaptation and after documenting the survival rate, plants were transferred to the greenhouse. Final acclimatization was carried out in the Elin Pelin field plots (640 m altitude, April 2022).

#### 4.1.5. Conditions for In Vitro Cultures 

In vitro materials were cultivated in a growth room with artificial illumination (lamps type fluorescent-FL^−40^ W^−1^, Svetlina Ltd., Stara Zagora, Bulgaria) under a 16 h photoperiod at 18–21 °C, with a photon flux density of 40 μM m^−2^ s^−1^ for stem tips cultivation and shoot formation and 20 μM m^−2^ s^−1^ for seed germination, root initiation and maintenance.

### 4.2. Phytochemical Analysis

#### 4.2.1. Extraction of Polyphenols and Flavonoids

Plant materials were dried in shade at room temperature and grinded with a laboratory mill (Optimum RK-0150, Warsaw, Al. Witosa 31/22, Poland). For the extraction, 0.5 g of the dried powder was added to 40 mL of 70% (*v*:*v*) ethanol solution in water. Extraction was conducted with a magnetic stirrer at room temperature for 1 h. The slurry was centrifuged (6000× *g*) and supernatants were collected. Plant extracts denoted as IVS–in vitro-propagated shoots grown on Z_1_I_0.1_ medium; FGP–in vitro-obtained field-grown plants; ICP–in situ-cultivated plants, provided by “EkoBilko” Ltd., (Trigrad town Bulgaria) were used for total polyphenol and flavonoid content and antioxidant activity determination.

#### 4.2.2. Preparation of Freeze-Dried Extracts

Five grams of the dried powder was added to 200 mL water (90 °C) and incubated for 15 min. The slurry was centrifuged (6000× *g*) (MPW-260R, MPW Med. Instruments, Warszawa, Poland) and supernatants were collected and freeze dried for 96 h in an Alpha 1–4 LD plus laboratory freeze drier (Martin Christ Gefriertrocknungsanlagen GmbH, Osterode am Harz, Germany) (Table S1). Freeze dried extracts were used for determination of antitumor activity.

#### 4.2.3. Determination of Polyphenol Content 

Total polyphenols were determined according to the method of Singleton and Rossi [68] and gallic acid as a calibration standard. Results were expressed in mg gallic acid equivalents (GAE) per 100 g dry weight (DW) ± SD.

#### 4.2.4. Determination of Flavonoid Content 

The total flavonoid content was determined according to Chang et al. [69] using AlCl_3_ reagent and a calibration curve built with rutin (10–200 mg/L) as a standard compound. Results are expressed as mg rutin equivalents (RE) per 100 g DW ± SD.

#### 4.2.5. Antioxidant Activity Assays

Oxygen radical absorbance capacity (ORAC) and hydroxyl radical averting capacity (HORAC) were measured according to the methodology of Ou et al. and Ou et al. [70,71] and details reported by Denev et al. [72]. Results are presented as micromole Trolox equivalents (µmol TE) and micromole gallic acid equivalents (µmol GAE) per gram dry plant material for ORAC and HORAC, respectively. The measurements were carried out on a FLUOstar OPTIMA plate reader (BMG Labtech, Ortenberg, Germany). All chemicals for phytochemical analyses were of analytical grade. Folin–Ciocalteu’s phenol reagent was purchased from Merck (Darmstadt, Germany). Gallic acid, 2,2-azobis-(2-amidinopropane) dihydrochloride (AAPH), 6-hydroxy-2,5,7,8-tetramethylchroman-2-carboxylic acid (Trolox), fluorescein disodium salt, quercetin-3-rutinoside (rutin) and gallic acid were delivered from Sigma-Aldrich (Steinheim, Germany). All other reagents and solvents used purchased from local distributors.

### 4.3. Antitumor Activity

#### 4.3.1. Cell Lines

BALB/3T3, clone A31–mouse embryonic fibroblasts, HeLa–human cervical carcinoma, HT-29–human colorectal carcinoma and MCF-7 –human mammary carcinoma were obtained from the American Type Culture Collection (ATCC) and were grown in Dulbecco’s modified Eagle medium (Sigma-Aldrich, Steinheim, Germany) supplemented with 10% fetal calf serum (Gibco, Lofer, Austria), 2 mM glutamine and the antibiotics penicillin 100 U mL^−1^ and streptomycin 100 µg mL^−1^ at 37 °C and 5% CO_2_.

#### 4.3.2. MTT Dye Reduction Test

The cytotoxic and antiproliferative potential of the extracts prepared from in situ-cultivated plants (ICP), field-grown in vitro-obtained plants (FGP) and in vitro shoots (IVS) *Sideritis scardica* samples was evaluated using the MTT-test following the standard procedure [73]. The human tumor cell lines HeLa, HT-29 and MCF-7 were used as in vitro cancer models and BALB/3T3 cells were used as a non-tumor control. Cell cultures in the exponential phase of growth were trypsinized and seeded in 96-well microtiter plates (100 μL/well) at a density of 1 × 10^5^ cells/mL. The cells were allowed to adhere for 24 h and were then treated with four concentrations of each *S*. *scardica* extract ranging between 125 µg/mL and 1000 µg/mL (six replicates/concentration). The control and treated cells were incubated for 24 and 48 h in an incubator at 37 °C and 5% CO_2_. The treatment medium was replaced with DMEM containing 0.5 mg/mL MTT and the cells were incubated for 3 h. The MTT solution was removed from the plates and the formed formazan crystals were dissolved in DMSO: 96% ethanol (1:1 *v*/*v*) solution. Optical density of the samples was measured using a microplate reader (TECAN, Sunrise TM, Groedig/Salzburg, Austria) at 540 nm. The experiments were performed in three replicates.

#### 4.3.3. Cytomorphological Analysis

Fluorescent microscopy study of HeLa cells treated with IVS extract was performed to analyze the morphological changes induced in the cancer cells. HeLa cervical carcinoma cells were seeded at a density of 1 × 10^5^ cells/well and were grown overnight on glass coverslips placed on the bottom of 24-well tissue culture plate in growth medium DMEM, at 37 °C and 5% CO_2_. The following day, the cells were treated with IVS extract, applied at a concentration approximating the IC_50_ value established by the MTT test performed at the 48th h. After 48 h exposure to the tested extract, two fluorescent staining methods were applied: DAPI staining and dual live/dead staining with acridine orange/ethidium bromide (AO/EB). The stained control and IVS-treated cells were immediately observed using a fluorescence microscope (Leica DM 5000B; Wetzlar, Germany).

### 4.4. Statistical Analysis

Statistical analysis on the in vitro propagation experiments was performed using one-way analysis of variance (ANOVA) for comparison of means, and Fisher’s least significance difference (LSD) test to calculate significant differences (Statgraphics Plus, version 5.1 for Windows). Results are presented as means ± standard deviations (SD). All experiments were performed in three repetitions. Phytochemical and biological activity test analyses are performed using Microsoft Excel (Microsoft Office Profesional Plus 2019). Data are expressed as means ± standard deviations (SD). All experiments were repeated four times. A statistical evaluation of antitumor activity was performed through ANOVA (GraphPad PRISM, version 5) followed by Bonferroni’s post hoc test (GraphPad PRISM, version 5). Nonlinear regression (a curve fit analysis) was used to determine the relevant IC_50_ concentrations.

## 5. Conclusions

In conclusion, the present study describes a detailed and efficient protocol for the micropropagation of the *S. scardica* species to aid in the preservation and further medicinal use of the endangered plant. The elaborated improved protocol for the in vitro propagation of *S. scardica* produces plants that accumulate polyphenols, including flavonoids, in amounts comparable to those found in plants cultivated in their natural habitat. The higher content of polyphenols and flavonoids underlies the higher antioxidant activity of the field-grown in vitro-obtained plants, which—together with the antitumor effects of field-grown in vitro-obtained plants and especially of in vitro propagated shoots—suggests their use as an adjuvant therapy for cancer and other oxidative stress-related diseases.

## Figures and Tables

**Table 1:** Effect of plant growth regulators on micropropagation of *Sideritis scardica*–Ist subculture.

Plant Growth Regulators [mg/L]	New Shoot Formation [%]	Number of Shoot per Explant	Shoot Height [cm]
Z_1_I_0.1_	100	6.0 ± 0.91 ^c^	2.4 ± 0.92 ^e^
Z_2_I_0.1_	80	11.0 ± 1.92 ^d^	2.0 ± 0.47 ^d^
K_1_I_0.1_	80	5.1 ± 1.29 ^b^	0.8 ± 0.15 ^bc^
B_1_I_0.1_	100	6.0 ± 1.33 ^c^	0.5 ± 0.11 ^ab^
K_1_N_0.1_	60	4.1 ± 1.16 ^a^	1.1 ± 0.39 ^c^
T_1_N_0.1_	80	5.0 ± 1.45 ^b^	0.4 ± 0.14 ^a^
B_1_N_0.1_	100	3.6 ± 0.99 ^a^	2.3 ± 0.85 ^de^
LSD	-	0.83	0.33

The data are presented as means of 40 shoots per medium variant ± standard deviation (SD). Different letters indicate significant differences assessed through a Fisher LSD test (*p* ≤ 0.05) after performing one-way ANOVA analysis.

**Table 2:** In vitro rooting of *Sideritis scardica* (1 month after transfer).

Nutrient Medium	Root Induction [%]	Number of Roots per Explants	Root Length [cm]
1/2MS + 0.1GA_3_ + 0.5 IAA	15	3.0 ± 0.79 ^a^	1.6 ± 0.52 ^c^
1/2MS + 0.6 IAA	60	4.0 ± 0.97 ^b^	2.5 ± 0.54 ^d^
1/2MS + 0.8 IAA	15	4.0 ± 0.85 ^b^	1.8 ± 0.48 ^c^
1/2MS + 1.0 IAA	15	3.0 ± 072 ^a^	1.3 ± 0.44 ^b^
1/2MS + 1.5 IAA	50	3.0 ± 0.85 ^a^	0.8 ± 0.23 ^a^
LSD	-	0.53	0.28

The data are presented as means of 40 shoots per medium variant ± standard deviation (SD). Different letters indicate significant differences assessed through a Fisher LSD test (*p* ≤ 0.05) after performing one-way ANOVA analysis.

**Table 3:** Total polyphenol and flavonoid content and antioxidant activity of in vitro-propagated shoots, in vitro-obtained field-grown and in situ-cultivated *Sideritis scardica* plants.

Sample	Total Polyphenols, mg GAE/100g DW	Total Flavonoids, mg RE/100g DW	ORAC, µmol TE/g DW	HORAC, µmol GAE/g DW
ICP	3563.5 ± 52.8 ^b^	927.9 ± 23.4 ^b^	939.9 ± 52.4 ^b^	268.2 ± 5.3 ^b^
FGP	3929.1 ± 112.2 ^c^	1024.3 ± 31.1 ^c^	1211.6 ± 27.3 ^c^	307.7 ± 4.5 ^c^
IVS	2404.6 ± 30.7 ^a^	359.1 ± 14.2 ^a^	821.1 ± 19.6 ^a^	198.6 ± 5.6 ^a^
LSD	147	23	72	204

Legend: ICP—in situ-cultivated plants; FGP—in vitro-obtained, field-grown plants; IVS—in vitro-propagated shoots. The data are presented as means of three samples ± standard deviation (SD). Different letters indicate significant differences assessed using the Fisher LSD test (*p* ≤ 0.05) after performing ANOVA multifactor analysis.

**Table 4:** Inhibitory concentrations (IC_50_) of extracts from in situ-collected, in vitro-obtained field-grown and in vitro shoots of *Sideritis scardica* samples on BALB/3T3, HeLa, HT-29 and MCF-7 cell lines.

IC_50_ µg/mL	Balb/c 3T3		HeLa		HT-29		MCF-7	
	24 h	48 h	24 h	48 h	24 h	48 h	24 h	48 h
ICP	>1000	>1000	>1000	>1000	>1000	>1000	709.1	719.2
FGP	957.1	648.3	910.7	907.5	>1000	>1000	633.5	652.0
IVS	>1000	>1000	>1000	629.2	>1000	>1000	>1000	>1000

Legend: ICP—in situ-cultivated plants; FGP—in vitro-obtained, field-grown plants; IVS—in vitro-propagated shoots.

## Data Availability

All data is comprised in the manuscript.

## Supplementary Materials

The following supporting information can be downloaded at: https://www.mdpi.com/article/10.3390/plants12233924/s1, Table S1: Total polyphenol and flavonoid content and antioxidant activity of freeze-dried extracts from in vitro-propagated shoots, in vitro-obtained field-grown and in situ-cultivated *Sideritis scardica* plants (see codes in Table 3).
