# Supplementary material for: Antioxidant and Antitumor Potential of Micropropagated Balkan Endemic Sideritis scardica Griseb
Source: Plants (Basel). 2023 Nov 21;12(23):3924. doi: 10.3390/plants12233924 (PMC10707862; doi:10.3390/plants12233924)
Supplement: Supplementary file 1 [file plants-12-03924-s001.zip › plants-2720273-supplementary.pdf]

Supplementary Table S1. Total polyphenol and flavonoid content, and antioxidant activity of freeze-dried extracts from *in vitro* propagated shoots, *in vitro* obtained field grown and *in situ* cultivated *Sideritis scardica* plants.

Sample	Total polyphenols, mg GAE/100g DW	Total flavonoids, mg RE/100g DW	ORAC, μmol TE/g DW	HORAC, μmol GAE/g DW
ICP	12095.8±1207.6^{b}	1902.5±229.3^{a}	2595.4±33.5^{b}	718.0±40.5^{b}
FGP	13853.4±627.3^{c}	3394.2±248.7^{b}	2708.4±73.7^{c}	838.4±19.0^{c}
IVS	7870.4±223.1^{a}	1600.9±12.2^{a}	1072.2±23.0^{a}	459.1±18.3^{a}
LSD	1591	188	97	56

Legend: ICP – *in situ* cultivated plants; FGP – *in vitro* obtained, field grown plants; IVS – *in vitro* propagated shoots. The data are presented as means of 3 samples ± standard deviation (SD). Different letters indicate significant differences assessed by Fisher LSD test (P≤0.05) after performing ANOVA multifactor analysis.
